# Does Total Hip Arthroplasty Influence Pelvic Version? A Retrospective Case Control Study Using the Sacro-Femoro-Pubic Angle in Osteoarthritis and Fracture Patients

**DOI:** 10.3390/medicina61081414

**Published:** 2025-08-05

**Authors:** Giuseppe Geraci, Alberto Corrado Di Martino, Enrico Masi, Alessandro Panciera, Chiara Di Censo, Cesare Faldini

**Affiliations:** 1 Ist Orthopaedic Department, IRCCS–Istituto Ortopedico Rizzoli, via Giulio Cesare Pupilli, 1, 40136 Bologna, Italy; giuseppe.geraci@ior.it (G.G.); enrico.masi@ior.it (E.M.); alessandro.panciera@ior.it (A.P.); chiara.dicenso@ior.it (C.D.C.); cesare.faldini@ior.it (C.F.); 2 Department of Biomedical and Neuromotor Science-DIBINEM, University of Bologna, 40126 Bologna, Italy

**Keywords:** total hip arthroplasty, pelvic version, sacro-femoro-pubic angle, pelvic tilt, spinopelvic alignment, complications

## Abstract

*Background and Objectives*: Spinopelvic alignment may affect the outcomes of total hip arthroplasty (THA), with pelvic version influencing the risk of mechanical complications occurring after surgery. On the other hand, THA surgery itself may contribute to the modification of pelvis version. The sacro-femoro-pubic (SFP) angle is measured on anteroposterior (AP) radiographs of the pelvis in a supine position, and is used to estimate pelvic tilt (PT), representative of pelvic version, which requires lateral views of the sacrum for its calculation; however, these X rays are not routinely performed in the preoperative setting of hip surgery. This study aims to analyze how THA determines changes in the pelvic version of operated patients; the SFP angle will be used to assess pelvic version on standard AP radiographs. *Materials and Methods*: This retrospective study included 182 consecutive patients undergoing THA for unilateral primary degenerative hip osteoarthritis (HOA-study group, *n* = 104) or femoral neck fracture (FNF-control group, *n* = 78) at the author’s institution. The SFP angle was measured on AP pelvic radiographs of the non-replaced hip preoperatively, postoperatively, and at the last follow-up. PT values were derived from SFP angles. Pre- and postoperative PT and its variations ΔPT were assessed. Study groups were compared in terms of native and postoperative variations of pelvic version. *Results*: The average absolute value of ΔPT was 2.99° ± 3.07° in the HOA group and 3.57° ± 2.92° in FNF group. There was no significant overall difference in preoperative or postoperative PT values between groups. In both groups, THA surgery led to a certain improvement, still not significant, in pelvic orientation, with FNF patients presenting a greater tendency toward retroversion. No significant differences in complication rates were found comparing patients with different pelvic orientations. *Conclusions*: THA can lead to a “normalization” of pelvic version in a certain number of patients with preoperative anteversion or retroversion. Although statistically non-significant, this observation may have clinical implications for spinopelvic balance and could support prioritizing THA in patients with concurrent spinal disease. Further research is needed to confirm these findings and to evaluate the long-term impact of THA on spinopelvic alignment.

## 1. Introduction

Total hip arthroplasty (THA) for the treatment of primary osteoarthritis (OA) is considered one of the most successful procedures in orthopaedic surgery [1], mainly thanks to its significant impact on relieving functional compromise and pain. Due to overall good surgical outcomes and the implementation of minimally invasive techniques, it is now considered a highly standardized procedure with reproducible results. It is estimated that approximately 80,000 THA procedures are performed annually in Italy, with implant survival rates exceeding 90% at 20 years [2].

Spino-pelvic motion is a relevant aspect determining the outcome of patients operated on THA: abnormal pelvic version and kinematics are related to mechanical complications, including impingement, instability, dislocation, and early wear of the components [3]. Understanding the interactions between the spine and pelvis is one of the critical times of modern preoperative evaluation to plan an effective surgical strategy and to reduce the risk of complications following THA [3]. Pelvic incidence (PI), pelvic tilt (PT), and sacral slope (SS) are radiographic parameters assessed in lateral spino-pelvic radiographs. Their values and changes in sitting and standing positions are informative about the anatomical and functional relation between the hip joint and the pelvis; however, lateral radiographs of the pelvis are not routinely performed before THA surgery [4].

Given the predominant use in clinical practice of anteroposterior (A-P) radiographs in a supine position compared to lateral ones, the sacro-femoro-pubic (SFP) angle [5] has been established as a surrogate measurement of the sagittal orientation of the pelvis, being representative of the pelvic tilt (PT).

While the clinical utility of this angle is still debated [6], it could be extremely valuable as a research tool; the SFP angle could be an effective instrument in identifying possible variations in pelvic version within the same patient before and following THA. Being the SFP angle non routinely evaluated in the clinical setting, it requires clinical studies to support its widespread use.

Therefore, this study aims to analyse SFP angles (and the derived PT) on A-P radiographs:

1. To estimate pelvic version in patients undergoing THA with diagnosis of unilateral primary hip OA (HOA-study group) or femoral neck fracture (FNF-control group), assessing whether the pelvic version is influenced by THA performance, and if predictive factors of pelvic changes can be determined;
2. To assess any potential difference between study groups related to the primary disease (OA vs. NF);
3. To study whether changes in pelvic version after surgery can influence the surgical outcome in terms of the incidence of complications.

## 2. Materials and Methods

This is a monocentric, retrospective, case-control study. Patients were selected as part of a research project approved by the local ethics committee (code ANT-HIP/2021/Oss/IOR347/2021/Oss/IOR).

### 2.1. Patient Selection

Patients who underwent unilateral THA (at any age ≥ 18 years old at the time of surgery) between 1 January 2020, and 31 December 2023, at the authors’ institution, with a diagnosis of unilateral primary HOA (study group) or FNF (control group) were included. Although there are limitations and the results should be interpreted with caution, patients with FNF are representative of the population without HOA undergoing the same surgical procedure. The exclusion criteria included: THA for any other diagnosis (including bilateral hip disease), presence of a contralateral THA, missing or inadequate radiographic examinations, and incomplete clinical data.

All the THA surgical procedures were performed by different senior surgeons, and the same type of implant has been used for all surgical procedures: Versafit CC Trio (Medacta, Castel San Pietro, Switzerland) for the cup, a hydroxapatite-coated elliptical cup, and AMIS stem-H or AMIS stem-P (Medacta, Castel San Pietro, Switzerland) for the stem, a straight, triple tapered, hydroxyapatite-coated, not cemented stem.

Joint coupling components were Biolox Delta (CeramTec, Plochingen, Germany) ceramic-on-ceramic in 95.7% of hips, and ceramic-on-XLPE in 4.3% of patients.

Demographics and clinical and radiological records were collected from the institutional database; radiographic parameters were measured using the image management software “VuePacs Carestream V12.1.” For each patient, sex, age at the time of surgery, diagnosis leading to THA, and clinical data regarding any intraoperative and postoperative complications were collected.

**Figure 1:**
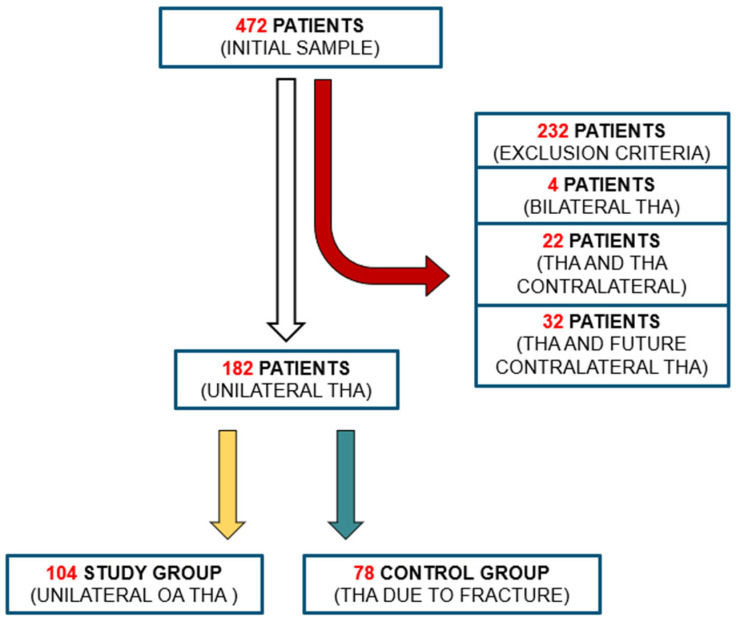
Flowchart outlining the process of inclusion and exclusion of patients into the study groups. Patients diagnosed with HOA were allocated to the study group, whereas those presenting with a FNF were assigned to the control group.

Initially, 472 patients were selected based on the inclusion criteria (Figure 1). The application of exclusion criteria reduced the study cohort to 182 patients who underwent unilateral THA in the absence of a contralateral implant: 104 had a diagnosis of unilateral primary degenerative arthritis (HOA) and 78 had a diagnosis of proximal femoral neck fracture (FNF).

### 2.2. Radiographic Evaluation

Standard AP pelvic radiographs were obtained using Carestream Health Inc., the picture archiving and communication system (PACS) available at our institution. Collected images were critically evaluated to verify their proper execution in terms of symmetry and horizontal alignment to the pelvis. Correct measurement of the SFP angle requires the pelvis to be horizontally aligned and as perpendicular as possible to the X-ray beam [5]. The radiograph should include all reference points necessary for angle measurement, including the superior surface of the sacrum, the center of rotation of the femoral head, and the upper aspect of the pubic symphysis. Additionally, the radiograph must capture the contralateral hip joint to facilitate SFP angle measurement. Radiographs that did not meet these criteria were excluded.

**Figure 2:**
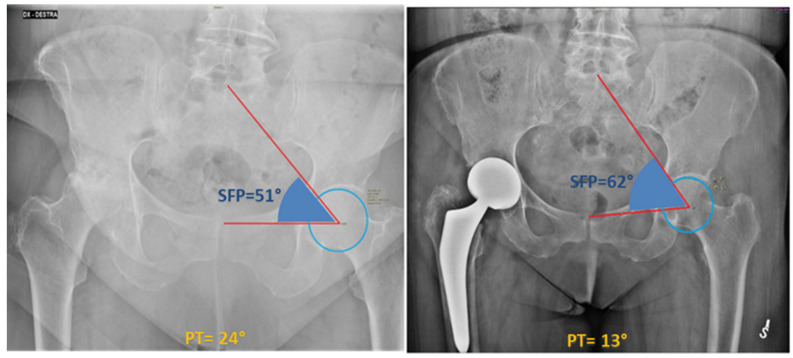
The SFP angle is measured on the coronal view of a standard anterior-posterior pelvic radiograph. The SFP angle, according to the description by Blondel et al. [5], is defined as the angle between the line passing through the midpoint of the upper sacral endplate, the centroid of one femoral head, and the upper midpoint of the pubic symphysis. This figure shows the different SFP angle between a preoperative (51°) and postoperative (62°) pelvis radiograph of a 77-year-old woman.

Sacro-femoro-pubic (SFP) angle was measured independently by two authors (G.G., E.M.) at the non-affected hip as shown in Figure 2 for each patient before surgery, immediately after surgery, and finally at the last available follow-up (FU) (average last follow-up time: 32.4 months). The angle was routinely measured on the non-prosthetic hip to assess any variations, as THA can shift the centre of rotation of the joint (often medially), thereby hindering the accurate measurement of the SFP angle: indeed, medializing the centre of rotation of the hip, as typically occurs in THA, would increase the SFP angle of the same hip, thereby simulating pelvic anteversion. Conversely, assessing the SFP angle on the contralateral hip inherently minimizes the risk of measurement bias. Excellent interobserver reliability was verified for the SFP angle (ICC superior to 0.9). Pelvic tilt (PT) was derived for each patient, according to the nearly-linear correlation with the SFP angle [5]: PT = 75 − SFP.

### 2.3. Clinical Data Collection

Specific mechanical and non-mechanical complications were collected regarding the prosthetic implant, and these were extracted from the institutional clinical database. Information gathered from follow-up visits and any new admissions for elective outpatient or emergency visits was recorded.

Following verification of the normal distribution of the PT data, the patients in the two groups (HOA, FNF) were categorized into three subgroups (neutral, anteverted, and retroverted pelvis) based on the distribution of their pelvic version values, using a threshold of ± 1SD [7].

### 2.4. Statistical Analysis

Descriptive statistics, including average, standard deviation, and range, were used to present the data. Normal distribution of the data was assessed using the Shapiro–Wilk test. Differences between groups were evaluated using Student’s *t*-test (paired or unpaired) or Chi-square test as appropriate, with statistical significance set at *p* < 0.05.

## 3. Results

Of the 182 patients selected according to the inclusion and exclusion criteria, 103 (56.6%) were female and 79 were men (43.4%). The mean age at surgery was 69 ± 12.34 years (range 23–95) and 91 THAs (50%) were implanted at the right limb and 91 (50%) at the left side.

Study group (HOA) included 104 patients (57.14%), while the control group (FNF) consisted of 78 (42.86%) proximal femur fracture patients. The OA group was composed of 56 women (53.85%) and 48 men (46.15%). The average age was 65.27 ± 12.84 years (range: 23–95). 53/104 implants (50.96%) were performed at the left limb and 51/104 (49.04%) at the right limb. The average follow-up period was 33.8 ± 14.1 months. Among the 78 patients of the FNF group, 47 were women (60.26%) and 31 were men (39.74%). The average age was 74 ± 9.66 years (range: 44 to 90). Forty THAs (51.28%) were implanted at the right hip and 38 (48.72%) at the left hip. The average follow-up period was 30.6 ± 12.8 months (Table 1).

The age at the time of surgery was significantly higher in the control group (*p* < 0.001). No significant differences between groups were observed when gender or FU was considered.

### 3.1. Is Pelvic Tilt Influenced by THA?

In the study group (HOA), the average preoperative PT was 17.10° ± 7.76° (range: −7.43°, 37.43°). The Shapiro–Wilk test confirmed a normal distribution of the data (*p* < 0.05).

Three groups of patients were formed according to PT: the grouping criteria were established considering our data distribution (specific cut-off values for the pelvic version are not validated by the current literature). The mean PT +/− 1 DS (7.76°) was considered as the median reference. Patients with a PT > 25° were included in the retroverted pelvis group (16 cases), those with PT between 9.5° and 25° were included in the normoverted group (73 patients), while those with PT < 9.5° were included in the anteverted group (15 cases).

The mean postoperative PT was 17.79° ± 7.86° (range: −6.04° to 50.43°). Using the same cut-offs as in the preoperative evaluation, postoperatively, 12 patients showed a retroverted pelvis, 79 normoverted pelvis, and 13 patients anteverted pelvis.

No significant differences between preoperative and postoperative PT were found (*p* = 0.522; paired Student’s t-test). The mean absolute value of |ΔPT| (postoperative PT–preoperative PT) was 2.99° ± 3.07° (range: 0° to 15.06°). Sixty-five patients (62.5%) showed a ΔPT > 0° (mean 3.00° ± 3.14°), indicating a retroversion of the pelvis, 39 patients (37.5%) showed a ΔPT < 0° (mean −3.15° ± 2.99°), indicating an anteversion of the pelvis. Moreover, 12 patients (11.54%) exhibited an absolute |ΔPT| > 5° between preoperative and postoperative measurements.

No significant differences were observed regarding age at the time of surgery or gender comparing patients with retroversion (ΔPT > 0°) or anteverversion (ΔPT < 0°) of the pelvis following THA.

**Figure 3:**
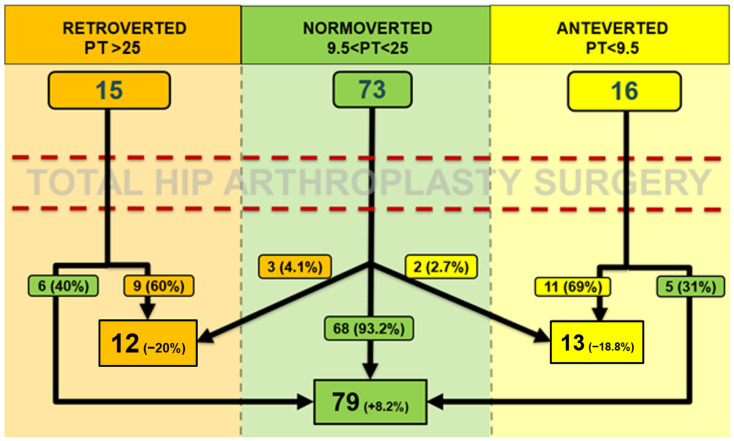
Comparing preoperative and postoperative assessments, a modification of pelvic orientation (exceeding cut-off values) was observed in 16 out of 104 patients (15.38%), 8 of which involved a shift towards retroversion (50%) and eight towards anteversion (50%). Specifically, among the eight cases of retroversion, five resulted from a shift from anteversion to a normoverted pelvis, while three shifted from a normoverted to a retroverted pelvis. Of the eight cases of anteversion, six involved a shift from retroversion to a normoverted pelvis, while two shifted from a normoverted to an anteverted pelvis. Furthermore, among the 15 patients who had a retroverted pelvis preoperatively (PT > 25°), 6 became normoverted postoperatively (40%); while of the 16 patients who had an anteverted pelvis preoperatively (PT < 9.5°), 5 became normoverted postoperatively (31.25%). Conversely, among the 73 patients with a normoverted pelvis preoperatively (9.5° < PT < 25°), 68 maintained a normoverted pelvis postoperatively (93.15%), while 5 patients (6.85%) transitioned to anteversion (*n* = 2) or retroversion (*n* = 3).

A comprehensive graphical representation of pre and postoperative pelvic orientation changes is depicted in Figure 3. Despite the varying number of patients in each group, no significant differences were observed in pre- and postoperative comparisons (*p* = 0.643).

In the control group (FNF), the average preoperative PT was 16.14° ± 7.72° (range: 2.49–40.38°). The Shapiro–Wilk test confirmed a normal distribution of the data (*p* < 0.05).

Three groups of patients were formed according to PT. The average PT +/− 1 DS (7.72°) was considered as the median reference. Patients with a PT > 24° were included in the retroverted pelvis group (10 patients), those with PT between 8.5° and 24° were included in the normoverted group (55 patients), then those with PT < 8.5° were included in the anteverted group (13 patients).

The mean postoperative PT was 17.83° ± 7.46° (range: 1.38° to 34.95°). Using the same cut-offs as in the preoperative evaluation, patients were similarly grouped in the postoperative period: 16 patients showed a retroverted pelvis, 54 patients were normoverted and 8 patients were anteverted.

No significant difference between preoperative and postoperative PT was observed (*p* = 0.165). The mean absolute value of │ΔPT│ (postoperative PT–preoperative PT) was 3.57° ± 2.92° (range: 0.09° to 15.52°), with 53 patients (67.95%) exhibited a ΔPT > 0° (mean 3.88° ± 3.26°), indicating retroversion of the pelvis, while 25 patients (32.05%) showed a ΔPT < 0° (mean −2.93° ± 1.95°), indicating anteversion of the pelvis. Additionally, 19 patients (24.36%) demonstrated an absolute │ΔPT│ > 5° between preoperative and postoperative measurements.

No significant differences were found regarding age at the time of surgery or gender comparing patients with pelvis retroversion (ΔPT > 0°) or anteversion (ΔPT < 0°) after THA.

**Figure 4:**
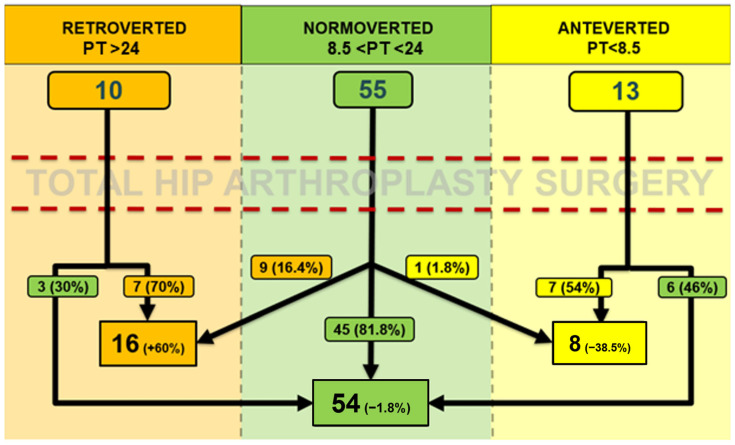
Comparing the preoperative and postoperative measurements, pelvic orientation variation (exceeding cut-off values) was observed in 19 out of 78 cases (24.36%), 15 of which involved a shift towards retroversion (78.95%) and 4 towards anteversion (21.05%). Specifically, of the 15 cases of retroversion, 6 resulted from a shift from anteversion to normoversion, while 9 shifted from normoversion to retroversion. Of the four cases of anteversion, three resulted from a shift from retroversion to normoversion, and one shifted from normoversion to anteversion. Additionally, among the 10 patients who had a retroverted pelvis preoperatively (PT > 24°), 3 became normoverted postoperatively (30%); among the 13 patients with an anteverted pelvis preoperatively (PT < 8.5°), 6 became normoverted postoperatively (46.15%). Conversely, of the 55 patients with a normoverted pelvis preoperatively (8.5° < PT < 24°), 45 maintained a normoverted pelvis postoperatively (81.82%), while 10 patients (18.18%) transitioned to anteversion (*n* = 1, 1.8%) or retroversion (*n* = 9, 16.4%).

A comprehensive graphical representation of pre- and postoperative pelvic orientation changes is depicted in Figure 4. No significant differences were observed in pre- and postoperative comparisons (*p* = 0.274).

### 3.2. Are There Differences Between HOA and FNF?

The preoperative PT of the study group did not significantly differ from that of the control group (Student’s *t*-test; *p* = 0.409). Similarly, postoperative PT did not significantly differ between groups (*p* = 0.9724), and the absolute ΔPT value of the study group did not significantly differ from the control group (*p* = 0.1995) (Table 2).

Considering the number of patients who showed retroversion (ΔPT > 0°) or anteversion (ΔPT < 0°) of the pelvis following THA, no significant differences were found (*p* = 0.4682) (Chi-square test) between groups. However, a higher number of patients in the FNF group (n.15, 19.23% vs. n.8, 7.69%) (*p* = 0.020) showed a tendency towards pelvis retroversion. This observation appears significative only when preoperative normoverted patients who become retroverted after THA surgery were considered (16.4% FNF vs. 4% HOA) (*p* = 0.041).

### 3.3. What Impact Does the Pre and Postoperative Pelvic Version Have on Complications?

After an average FU of 32.4 months (±13.5), an intraoperative and early postoperative complication rate of 5.5% (10/182) was observed. Of these events, 6 (60%) occurred in HOA and 4 (40%) in FNF patients. Specifically, complications in the HOA group included three periprosthetic fractures stabilized with metal wiring, one superficial infection, one symptomatic hip instability, and one dislocation managed by closed reduction (no recurrence). In the FNF group, complications included two deep infections managed by implant removal (Girdlestone procedure) and antibiotic therapy, one dislocation treated with closed reduction (no recurrence), and one periprosthetic fracture, stabilized with metal wiring.

Of the 10 complications, 7 occurred in male patients and 3 in female patients; 5 cases involved patients older than 70 years, and 5 cases involved patients younger than 70 years. In 8 cases, the patient had a normoverted pelvis both before and after surgery; in one case, the patient had a retroverted pelvis preoperatively and a normoverted pelvis postoperatively (periprosthetic fractures), and in one case, the patient had an anteverted pelvis both before and after surgery(dislocation).

No significant differences in complication rates were observed among patients with anteverted, normoverted, or retroverted pelvises, either within each study sample or when comparing the two samples.

## 4. Discussion

In the current study, the impact of THA on pelvic version was investigated using SFP (and derived PT) as a reference parameter, comparing two groups of patients with different diagnoses: those with unilateral primary hip OA (HAO study group) and those with fracture of the proximal femur (FNF control group). The study demonstrated a trend towards a pelvis normoversion after THA in patients with preoperative anteversion or retroversion. This trend was evident but non-significant in both the study and the control group. While no significant differences in pre- and postoperative PT were found comparing the two groups, a greater tendency towards retroversion was noted in the control group. Furthermore, no significant differences in complication rates were identified between patients with anteverted, normoverted, or retroverted pelvis, either within each group or in a comparative analysis among groups.

Several studies investigated the changes in pelvis orientation after THA. Grammatopoulos et al. [8] analyzed a cohort of 250 patients undergoing unilateral THA for primary OA. Lumbar lordosis (LL), pelvic tilt (PT), sacral slope (SS), and proximal femoral angle (PFA) on AP and lateral radiographs were collected in seated and standing positions before, one week, and one year after surgery, to assess the postoperative changes and identify predictive factors indicative of greater spinopelvic variability. The results of their study are comparable with our findings, since most patients (75%) do not undergo significant changes in spinopelvic radiographic parameters in the standing position one year after THA, demonstrating that the impact of THA does not significantly influence spinopelvic biomechanics.

Maratt et al. [9] collected PI and PT on LL radiographs in the standing position on a cohort of 138 patients undergoing unilateral THA [9]. Measurements were performed before and 7 weeks after surgery to assess postoperative changes and to measure the corresponding changes in acetabular cup orientation. The authors found an average ΔPT value of −0.3° ± 3.6° (range −9.6° to 13.5°), and they did not find a significant difference comparing preoperative and postoperative pelvis change (*p* = 0.395). Nineteen patients (14%) had a postoperative PT change of more than 5°, and only in one was the postoperative PT change more than 10°. It should be noted that Maratt et al. defined PT as the angle between the anterior pelvic plane (APP) and the coronal plane, while in the current study, PT was defined as the angle between the coronal plane and the line passing through the center of rotation of the femoral head and the midpoint of the superior sacral endplate (S1). However, despite these considerations, the average ΔPT values in the two studies remained similar (−0.3° vs. 1.12°).

Pelvis anteversion or retroversion may be the result of spine diseases or expression of different morphotypes [10]. Sagittal misalignment of the spine, as well as aging and spinal rigidity, often leads the pelvis to retrovert, with an increase in PT [5]. Aging is correlated with the physiological degeneration of lumbar disks, which results in a reduction in lumbar lordosis, with potential anterior sagittal imbalance. In such cases, compensatory mechanisms are employed to restore the sagittal vertical axis (SVA): pelvis retroversion with hip and knee flexion, which depends on the lumbopelvic complex and is reflected by the PI value [11]. On the other hand, primary hip OA leads to structural joint changes and mechanical limitations: these are associated with pain and determine changes in pelvic orientation with increased PT to compensate for loss of full hip extension, and potentially tilting laterally to compensate for leg-length discrepancies. Structural changes in the pelvis due to advanced osteoarthritis can affect the proximal adjacent segment, affecting the spine, which adopts compensatory mechanisms to better distribute loads.

The normalization of pelvic version after surgery observed in a certain number of patients with altered preoperative pelvic version can improve spinal alignment and clinical outcomes when spinal pathology occurs, in terms of pain and function [11]. For these reasons, data from this study, in accordance with current literature [12], seem to confirm that in patients with contemporary hip and spinal pathology, it is advisable at first to treat the hip joint and then consider spinal surgery, taking advantage of the potential improvement in global spinal balance provided by THA. This principle is supported by several studies. Zhang et al. [13] reported that patients with simultaneous diagnosis of hip OA and spinal disease are less likely to undergo spine surgery within 2 years if THA is performed first [13]. The authors compared a cohort of patients that underwent either hip or spinal surgery before the other: a more than fourfold difference in subsequent surgical intervention rates at 2 and 5 years was observed in favor of the “hip arthroplasty first” cohort. Similarly, several studies have demonstrated that THA yields better outcomes in patients with no history of lumbar spine arthrodesis or non-arthrodesis procedures.

Improving hip joint mobility and mechanics can enhance gait, posture, and reduce back pain by decreasing stress on the lumbosacral spine. Weng et al. [14] reported a significant rate (59%) of lumbar back pain relief following THA in a cohort of patients with simultaneous hip and spinal disease. Similarly, a study by Eguchi et al. [15] demonstrated that the visual analog scale (VAS) and Roland–Morris disability questionnaire for back pain significantly improved in patients with unilateral primary hip osteoarthritis after total hip arthroplasty. However, the same study reported no improvements in these metrics in patients with bilateral hip disease due to persistent dysfunction in the contralateral hip (i.e., flexion contracture, gait abnormalities, and leg length discrepancy). This underscores the intimate relationship between hip function and spinal symptoms and provides a potential explanation for higher rates of subsequent procedures when spinal surgery is performed first. Moreover, a registry study conducted by Di Martino et al. [12] underlines the hip surgery over spinal surgery at first, as patients who undergo lumbar fusion followed by total hip arthroplasty have an increased risk of mechanical complications following THA and a slightly higher risk of revision arthroplasty. Consequently, prioritizing THA in patients with concurrent spinal disease without neurological impairment may improve back pain and reduce the risk of subsequent spinal procedures.

This study shows that the same phenomenon of pelvic “normalization” tends to occur in patients with proximal femur fractures. However, unlike the sample of patients with unilateral primary hip OA, the sample of patients with proximal femur fractures, which consists of generally older patients, also exhibits a tendency towards pelvic retroversion. This is inevitably associated with the reduction in lumbar lordosis typical of older age, as previously discussed.

Regarding postoperative complications, six complications occurred in patients with unilateral primary hip OA, and four occurred in patients with proximal femur fractures. In 8 of the 10 cases, the patient had a normoverted pelvis both before and after surgery, in one case, the patient had a retroverted pelvis preoperatively and a normoverted pelvis postoperatively, and in one case, the patient had an anteverted pelvis both before and after surgery. Thus, complications occurred in a small number of cases relative to the total number of patients examined in this study, and they appear to occur regardless of preoperative and postoperative pelvic orientation, sex, and age of the patients, as well as the diagnosis of osteoarthritis or fracture. No individual predictive factors for a higher risk of postoperative complications were identified. It must be considered, however, that the limited number of patients is not adequate to provide significant results in this regard.

This study presents several limitations. First, the use of a coronal radiographic parameter like SFP, while a valuable alternative for assessing pelvic tilt, does not allow for perfect measurement and presents some margin of error. Nevertheless, according to Blondel et al. [5], the SFP is a parameter that can reliably estimate PT, as a statistically significant correlation between the two measurements has been found. The use of a single spinopelvic parameter as an indicator of spinopelvic biomechanics, while simplifying the analysis and comparison between study groups, does not strengthen the results of the study as would be the case if multiple spinopelvic parameters were examined. Furthermore, measurements were taken in the supine position and not in an upright position, preventing an adequate comparison of the SFP angle between the two different postures. Another limitation is that in the control group, preoperative measurements may be altered by an antalgic posture related to the fracture. Though it should be specified that the radiographs considered are not those performed in the emergency room, but those performed for adequate preoperative 2D planning of the THA procedure, usually acquired after achieving adequate pain control. It should also be considered that the cut-off values defining the different pelvic orientations differ between HAO and FNF patients. This choice was made specifically in consideration of possible differences in the distribution of observations. Nevertheless, the use of different cut-off values may lead to inaccurate interpretations when comparing the groups.

Another limitation of this study is the small number of patients in the study sample (*n* = 182), which does not allow for a proper evaluation of potential significant differences between the subgroups under examination. Finally, the absence of similar studies in the scientific literature prevents data aggregation and does not allow for adequate comparison of results.

## 5. Conclusions

THA may lead to an improvement in pelvic version in a portion of patients with altered preoperative pelvic version, potentially improving spinopelvic balance, both in patients with unilateral primary hip OA and in those with fracture of the proximal femur. This supports the thesis that in cases of concomitant hip and spinal pathology, it is advisable to perform THA first, potentially avoiding the need for spinal surgery later, or at least performing it under better spinopelvic alignment conditions.

No significant differences in pelvic version changes were observed between the two study groups. However, a tendency towards pelvic retroversion was noted in patients who underwent THA for fracture of the proximal femur. No predictive factors were identified for changes in pelvic orientation towards anteversion or retroversion. Clinical outcomes and complications in the study group did not show significant differences in relation to pelvic orientation in the two groups of patients.

In conclusion, hip arthroplasty has the potential to improve pelvic orientation, confirming its effectiveness as a treatment for primary hip osteoarthritis and medial fracture of the proximal femur with excellent clinical outcomes and low complication rates, even in patients with altered pelvic orientation.

## Figures and Tables

**Table 1:** Demographic data and differences between groups. In bold, statistically significant differences (*p* < 0.05).

	Study Group-HOA (n = 104)	Control Group-FNF (n = 78)	*p*-Value
**Age at surgery** **Mean ± SD (range)**	65.27 ± 12.84 (23–95)	74 ± 9.66 (44–90)	**<0.001**
**Male Gender (n (%))**	48 (46.15%)	31 (39.74%)	0.189
**Follow up**	33.8 ± 14.1 months	30.6 ± 12.8 months	0.108

**Table 2:** Differences between groups considering pre- and postoperative PT and absolute value of ΔPT. In bold, statistically significant differences (*p* < 0.05).

	Study Group-HOA (n = 104)	Control Group-FNF (n = 78)	*p*-Value
Preoperative PT (mean value ±SD)	17.10° ± 7.76°	16.14° ± 7.72°	0.409
Postoperative PT (mean value ±SD)	17.79° ± 7.86°	17.83° ± 7.46°	0.9724
Mean absolute value of ΔPT (mean value ±SD)	2.99° ± 3.07°	3.57° ± 2.92°	0.1995

## Data Availability

All collected data are reported in the current manuscript.

## Abbreviations

The following abbreviations are used in this manuscript:

FNF	femoral neck fracture
HOA	hip osteoarthritis
THA	total hip arthroplasty
SFP	sacro-femoral-pubic
PT	pelvic tilt
PI	pelvic incidence
